# Investigation of gadolinium doped manganese nano spinel ferrites via magnetic hypothermia therapy effect towards MCF-7 breast cancer

**DOI:** 10.1016/j.heliyon.2024.e24792

**Published:** 2024-01-19

**Authors:** M. Tahir, M. Fakhar-e-Alam, Muhammad Asif, M. Javaid Iqbal, Aoun Abbas, Mudassir Hassan, Javed Rehman, Qaisar Abbas Bhatti, Ghulam Mustafa, Asma A. Alothman, Saikh Mohammad

**Affiliations:** a Department of Physics, Government College University, Faisalabad, 38000, Pakistan; b Department of Zoology, Government College University, Faisalabad, 38000, Pakistan; c State Key Laboratory of Metastable Materials Science and Technology, and School of Materials Science and Engineering, Yanshan University, Qinhuangdao, 066004, China; d Department of Chemistry, Faculty of Sciences, Mohi-Ud-Din Islamic University Nerian Sharif, Azad Jammu & Kashmir, 12010, Pakistan; e Department of Physics, BZU, Multan, Pakistan; f Department of Chemistry, College of Science, King Saud University, Riyadh, 11451, Saudi Arabia; g MEU Research Unit, Middle East University, Amman, 11831, Jordan

**Keywords:** Magnetic spinal ferrites, Magnetic hyperthermia therapy, MRI, VSM and MCF-7

## Abstract

Magnetic spinel ferrite nanoparticles (MSF-NPs) are potential candidates for biomedical applications, especially in cancer diagnosis and therapy due to their excellent physiochemical and magnetic properties. In the current study, MSF-NPs were fabricated by sol-gel auto combustion method. The crystal structure and surface morphology were confirmed by X-ray diffraction (XRD) and scanning electron microscopy (SEM). The magnetic properties were studied by VSM (vibrating sample magnetometer). As increasing Gd^3+^ concentration, the saturation magnetization values decreased from (17.8-2.3) emu/g, while the coercivity decreased from (499-133) Oe at room temperature. Finally, the fabricated MSF-NPs were tested against anticancer activity by MTT assay. The IC_50_ = 21.27 μg/mL value was observed, showing the strong antiproliferative activity of these nanoparticles. These results suggested that the obtained MSF-NPs would be useful for remote-controlled hyperthermia therapy for cancer treatment and MRI application due to their excellent magnetic properties. These distinct properties make MSF-NPs most suitable for cancer treatment and bright Contrast Agents (T1-MRI).

## Graphical abstract

**Figure:**
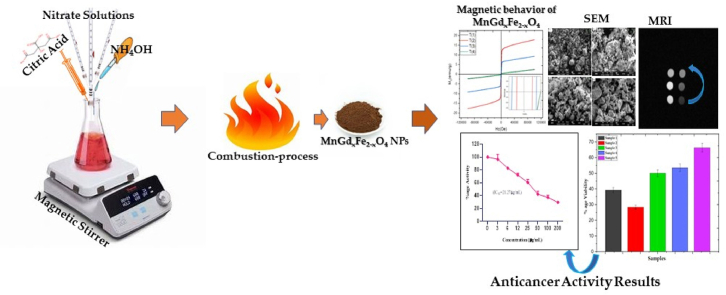

## 1 Introduction

Cancer is one of acute disease through which millions of people got causalities globally [1]. As per WHO report, 18.1 million new cancer cases and 9.6 million population deaths were reported in 2018. The populations attributed fraction (PAFs) in 2012, 2016 and 2017 were 1 %, 3–4% and 13 %, 4–5%, and 25 % due to obesity, UV light, infection, Alcohol and Tobacco, respectively. In 2018, lung, breast and colorectal cancer were the most common cases of cancer [9,10]. As per estimated cancer spread rate in 2035 about 24 million populations will be affected by malignancy [2]. There are many effective techniques for treatment modality of cancer disease i.e. chemotherapy (CT), photodynamic therapy (PDT) and photothermal therapy (PTT) but magnetic hyperthermia therapy (MHT) one of the sophisticated and noninvasive treatment modality which can overcome the interruption of nanoparticles and multidrug resistance (MDR) via proteins development during second mutation phenomena [[3], [4], [5]]. Among the different types efficient NPs, Magnetic spinal ferrites nanoparticles (MSF-NPs) have gained the great attention for their potential applications in various fields such as/including biomedical/biomedicine, catalysis, information technology, microwave gadgets, energy storage and sensors [[6], [7], [8]].

Magnetic Spinal ferrites nanoparticles (MSF-NPs) playing a vital role due to their small size, high surface to volume ratio, electrochemical, physicochemical, admirable biocompatibility, Specific targeting capability and owing magnetic properties and their auspicious biomedical applications such as hyperthermia [16], contrast enhancement agent in MRI [15], dynamic/immobilization agent in drug delivery and release [7,8]. Manganese gadolinium iron oxides nanoparticles (MnGdFe_2_O_4_ NPs) have supreme characteristics to biotechnology for their facial fabrication, stability, biosafety [[11], [12], [13], [14]]. There are some well-known methods to prepare MnGdFe_2_O_4_ MNPs such as Hydrothermal, sol-gel auto combustion and coprecipitation methods [15,16]. The main advantage of the MSF-NPs-MHT approach is the deep penetration and selective killing of cancer cells without damaging healthy tissues, increase the intracellular hyperthermia and being used to treat breast, hepatocellular and prostate cancer [[17], [18], [19], [20], [21], [22]]. In general, MSF-NPs-MHT increases the tumor local temperature in the range of 43–46 OC, thereby altering cancer cell physiology and leading to their apoptosis/necrosis [13,14]. There are fabulous conclusive properties of spinal ferrites, however still major enhancement and treatment morality and many unknown parameters has to be addressed [23]. In general, Magnetic hyperthermia (MH) therapy mainly involves increasing the local temperature of the tumor within the range of 43–46 °C, which results in altered cancer cell physiology, which ultimately causes their apoptosis/necrosis. It is imperious to provide sufficient heat to the entire tumor mass without affecting the healthy tissues. Thus, a strict limit in H and f of AMF is imposed where H × f < 5 × 10^9^ for biomedical reasons [24]. Magnetic hyperthermia, drug delivery, and magnetic resonance imaging (MRI) have been investigated using MnGdFe_2_O_4_ nanoparticles. When Magnetic MSF-NPs are exposed to an alternating magnetic field, they generate heat, which can be used for selective death of cancer cells, apoptosis, and inhibition of cancer cell proliferation [25].

MRI is an important non-invasive technique for the diagnosis and treatment evaluation of various diseases [26]. The usage of magnetic spinal ferrites nanoparticles (MSF-NPs) as contrast agents for magnetic resonance imaging (MRI) has enhanced the excellence of their results and the combination of different concentrations of manganese with magnetic nanoparticles for imaging, diagnosis and hyperthermia therapy [27]. Moreover, Magnetic MSF-NPs generate heat under alternating magnetic field (MF), which is very significant for cancer treatment in combination with photodynamic, radiotherapy and chemotherapy [28]. A strong MF generates a prevailing magnet inside the MRI that is used to line up the protons in the body, also MF produce by rotational and orbital motion of protons and Spin magnetization of polarized protons provides a central role in MRI images regardless of longitudinal (T1) and transverse (T2) relaxation rates [29]. To the best of our knowledge, the novelty of the research is that it is the first study to validate/demonstrate the anticancer and magnetic resonance imaging (MRI) efficacy of magnetic spinel ferrite nanoparticles (MSF-NPs). We recommend further in vivo anticancer such as (controlled Hyperthermia therapy, drug delivery) and MRI studies of magnetic spinal ferrite nanoparticles (MSF-NPs).

## 2 Materials and method

### 2.1 Chemicals

Manganese chloride tetra hydrate (MnCl_2_.4H_2_O), Iron nitrate [Fe (NO_3_)_3_.9H_2_O], Gadolinium nitrate hexahydrate [Gd (NO_3_)_3_.6H_2_O)] and ammonia Hydro oxide (NH_4_OH) were purchased from Merck (US). Additionally, the fetal bovine serum (FBS), antibiotics (100 μg/ml streptomycin and penicillin), DMEM (Dulbecco's Modified Eagle Medium) and dimethyl sulfoxide (DMSO) were purchased from Thermo fisher scientific.

### 2.2 Synthesis of nanomaterials

The Gadolinium Doped Manganese Iron Oxide Nanocomposites was synthesized via Sol-gel auto combustion method at different composition MnGd_x_F_2-x_O_4_ (x = 0.10, 0.15, 0.20, 0.25). The required composition was calculated with formula MnGd_x_F_2-x_O_4_. After calculation, all materials were added into a beaker by selecting 4.5136 g/mol of MnGdFeO_4_ into 100 ml of deionized water. First of all, sonication process was done continuously of about 30 min. Meanwhile, the beaker was allowed to vigorously stirring for 2 h. The stirring process was continued until the solution attained 7 pH. The pH was controlled with an ammonia solution. The main requirement of the synthesized material pH is equal to 7. So, all processes were at 100 °C. When the solution converts to gel, the temperature increases from 100° centigrade to 200° centigrade. After all the gel converts to ash. The ash placed into a mortar and pestle for grinding.process.. The final powder was incubated in a muffle furnace at 800 °C for 8 h. After the sintering process, the materials were analyzed for further characterization and mentioned biomedical activities.

### 2.3 Characterization of nanomaterials

X-ray diffraction analysis was carried out using a (“D8 Advance Bruker, Germany” with λ = 0.154 nm of Cu Kα radiation) x-ray diffractometer. The “NOVA NANOSEM 430” scanning electron microscope was used to analyze the morphology of the synthetic materials. A vibrating sample magnetometer EZ9 VSM was used to measure the magnetic characteristics of the synthesized materials at room temperature.

### 2.4 Assessment of anticancer activity

#### 2.4.1 Cell culturing

Human breast cancer (MCF-7) cell line were obtained from the Molecular and Cell Biology laboratory at the Department of Zoology, Government College University Faisalabad. The MCF-7 cells were cultured in 96 well plate in DMEM (Dulbecco's Modified Eagle Medium) supplemented with 10%FBS (fetal bovine serum), antibiotics (100 μg/ml streptomycin and penicillin) and cells were incubated at 37 °C with 5 % carbon dioxide (CO_2_) in humidified environment. For more details see our previous published paper protocol [30].

#### 2.4.2 MTT assay

The in-vitro cytotoxicity of Gadolinium doped manganese iron oxide nanocomposites was tested on MCF-7 cell line via MTT assay. MCF-7 cells were seeded in 96 well plate and further incubated for 48 h at 37C^0^. Then, the cells were cultured at various concentration of nanocomposites for 48 h to be treated. 10 μL of MTT reagent was added after 48 h and further incubated at 37 ^°^C for 4 h. Finally, added the DMSO (150 μL) to dissolve the formazan crystal and absorbance was calculated by microplate reader [[30], [31], [32]].

The absorbance of the treated and controlled cells was measured by given Eq. (1).

(1) $$\%\;Inhibation=\frac{Abs\left(control\right)-Abs\left(treated\right)}{Abs\left(control\right)}\times 100\%$$

#### 2.4.3 Magnetic hyperthermia and specific absorption rate (SAR) experiment

In the MH experiment, the self-heating properties of the samples were evaluated by subjecting them to an alternating magnetic field (AMF) of 30 mT in deionized water. The frequency was maintained at a constant value of 850 KHz for all the different samples (T1, T2, T3, T4, and T5). Furthermore, the specific sample T2 was tested at different concentrations, while keeping the frequency constant under the influence of 30 mT AMF.

The SAR measurements were conducted using national equipment, operating at a frequency of approximately 100 kHz. Various field strengths ranging from 2 to 42 mT were employed. In summary, SAR measurements were carried out by connecting a signal generator (with an input signal of 7.2 Vpp) to a HAS 4014 linear amplifier [58].

#### 2.4.4 T1 MRI contrast agent

The Magnetic resonance imaging (MRI) experiments were performed using a 1.5T scanner with standard mouse and collie size. The Magnetic Spinal ferrites nanoparticles (MSF-NPs) were used with different concentration of Gd & Fe (0–0.5 mM). T1 bright images were attained using different parameters such as repetition time (TR = 5000 ms), multi echo/fast spin echo, Number of statistics = 20, N_A_ (number of averages = 2), slice width = 5 mm echo time (TE = 7 ms) and relaxation enhancement. The T1 map's algorithms produced a strong signal (Bright contrast) [33].

## 3 Result and discussion

### 3.1 X-ray diffraction study

**Fig. 1:**
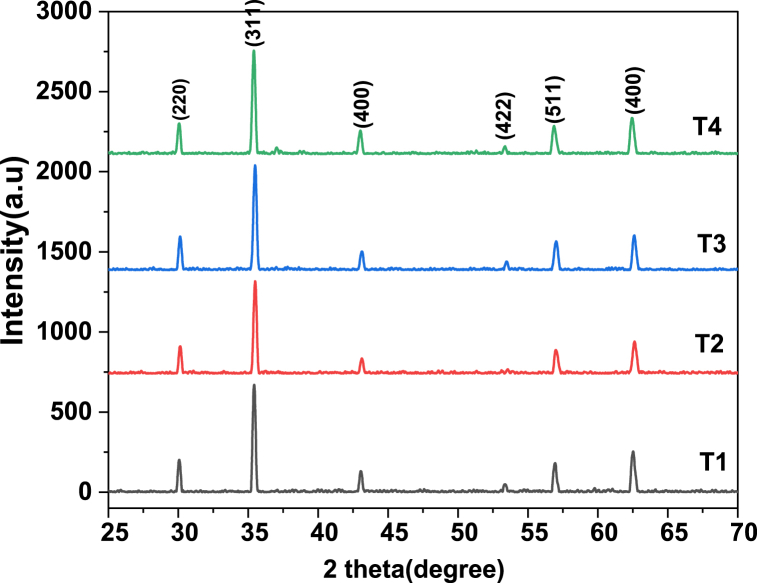
Xrd patterns of MnGd_x_Fe_2-x_O_4_ at (x = 0.10, 0.15, 0.20 and 0.25).

**Fig. 2:**
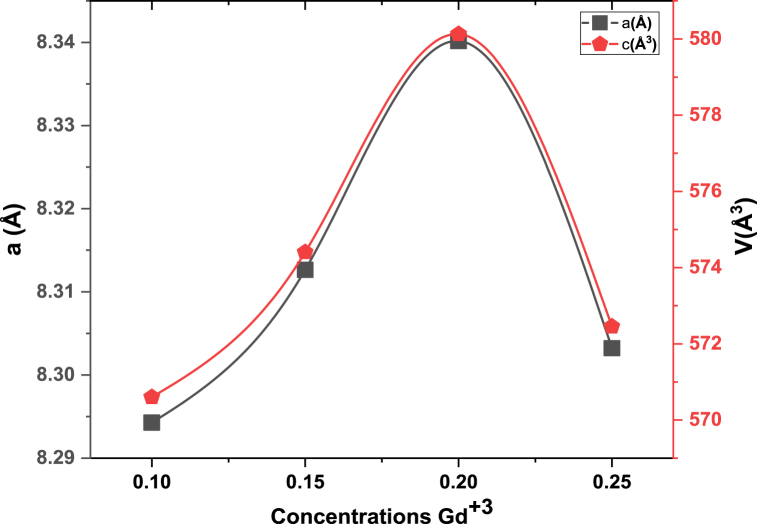
concentration Gd^3+^ versus lattice constant and unit cell volume.

**Fig. 3:**
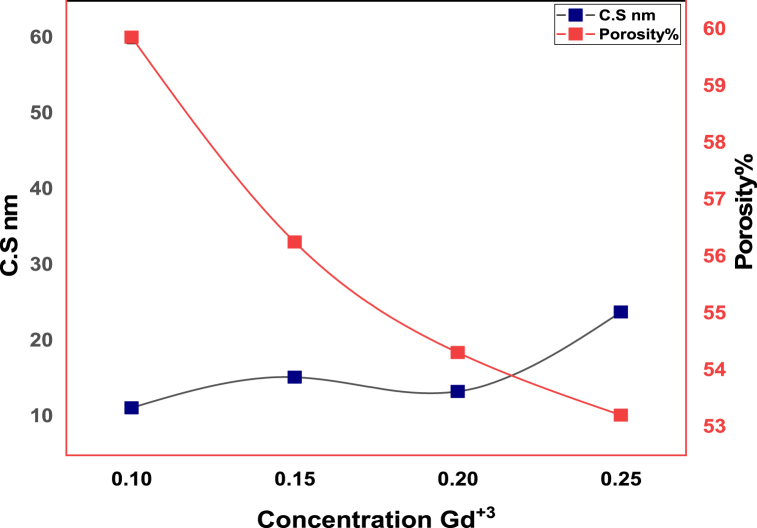
Concentrations Gd^3+^ Vs crystallite size (nm) and Porosity.

**Fig. 4:**
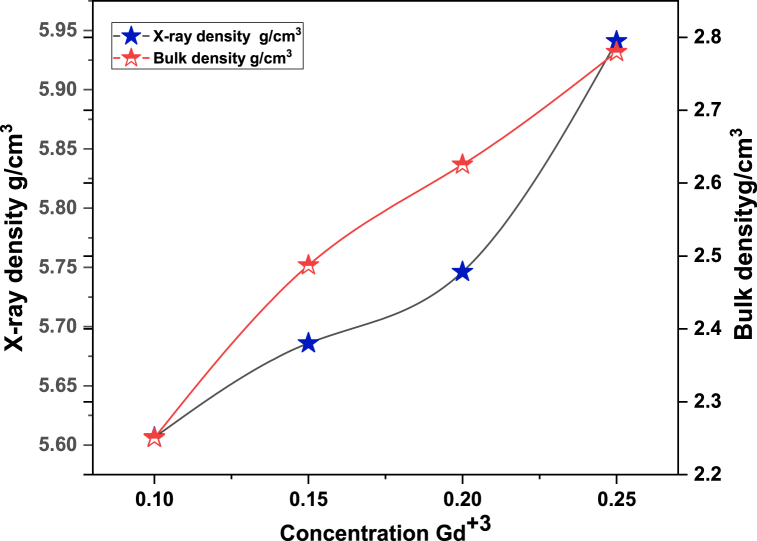
Concentration Gd^3+^Vs x-ray density and bulk density (g/cm^3^).

In Fig. 1 the XRD (x-ray diffraction) studied structural properties of synthesized material. Fig. 1 showed confirmation of a single phase and no undesired peaks were found. The behavior of cubical structure was explored and peaks were showed Fd-3m space group. These planes showed pattern confirm spinel ferrites. The required composition was MnGdxF2-xO4 (x = 0.10, 0.15, 0.20, 0.25). The brag's diffraction planes were (220), (311), (400), (422), (333), (440), and (511). An undesirable peak at ∼36.5° was observed in sample T4, this is due to air contamination. They crystallite size was calculated by Scherrer formula. The crystallite size was from 11.3 nm to 23.9. Fig. 2 shows that increasing the concentration of Gd^3+^ as also increase the crystallite size. The x-ray density and bulk density also increases as concentration Gd^3+^ increased. The bulk density ranged from 2.3 to 2.8 g/cm^3^, whereas the X-ray density ranged from 5.6 to 5.9 g/cm^3^. Fig. 3 shows that the crystallite size and other lattice parameters exhibit this behavior mainly due to the atomic and ionic radii of the rare earth elements that substitute for the iron ions. Similar results have already been reported in the literature [[34], [35], [36]]. As the contents Gd^3+^ ions enhanced the lattice parameters and volume of unit cell also increased. But other side the crystallite size and porosity of synthesized samples had opposite behavior. These changes were occurred due to different ionic radii of manganese and Gd^3+^ content. Finally, Fig. 4 shows that the bulk density and X-ray density of the fabricated samples displayed nonlinear behavior as the concentration of Gd^3+^ was increased [36,37]. These values were calculated by the given formulas.

(2) nλ=2dsinθ

(3) $$C.S=\frac{k\lambda }{\beta \;\cos\;\theta }$$

(4) $$a=d\;\sqrt{h^{2}+k^{2}+l^{2}}$$

(5) $$X-ray\;density=\frac{8M}{N_{a}*a^{3}}$$

(6) $$Bulk\;density=\frac{Mass}{\pi r^{2}h}$$

(7) $$\%Prosity=\frac{1-d_{b}}{d_{x}}\times 100\%$$

Table 1 depict the crystalline size (nm) and porosity (%) of sample T-1, T-2, T-3 and T-4. After data analysis it was investigated that crystalline size exists between 11.3 nm and 23.9 nm and respective porosity range (%) from 59.9 to 53.2 as shown above.

**Table 1:** Information regarding Lattice parameters, Crystallite size, Cell volume, X-ray density, Bulk density and Porosity of synthesized MnGd_x_Fe_2-x_O_4_ (x = 0.10, 0.15, 0.20, 0.25) at temperatures 800 °C for 8 h.

Sample code	*hkl*	2θ (degree)	CS (nm)	Lattice Constant (Ǻ)	Volume of unit cell (m^3^)	X ray Density (g/cm^3^)	Bulk density (g/cm^3^)	Porosity (%)
**T-1**	311	35.85	11.3	8.29	570.6	5.6	2.3	59.9
**T-2**	311	35.76	15.3	8.31	574.4	5.7	2.5	56.3
**T-3**	311	35.64	13.4	8.34	580.1	5.75	2.6	54.3
**T-4**	311	35.81	23.9	8.30	572.5	5.9	2.8	53.2

### 3.2 Morphological and texture analysis

**Fig. 5:**
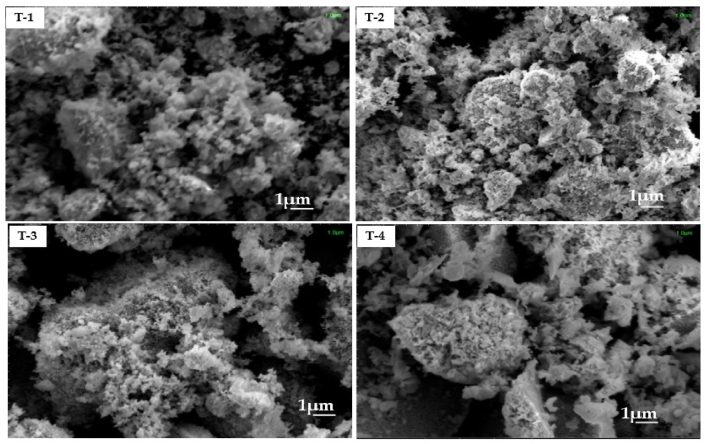
(T1, T2, T3, T4). shows the morphology of MnGd_x_Fe_2-x_O_4_ (x = 0.10, 0.15, 0.20 and 0.25) at 800 °C.

SEM analysis was performed to examine the surface morphology of the Magnetic spinel ferrite nanoparticles (MSF-NPs). Fig. 5 (T1, T2, T3 and T4) shows SEM images of nanoparticles with various Gd^3+^ concentrations (x = 0.10, 0.15, 0.20, and 0.25). As seen in Fig. 5 (T1, T2, T3 and T4), all of the samples had agglomerated and non-uniform spherical grains. Its means that the synthesis technique produced nanoparticles with an agglomerated spherically and non-uniform grain structure. SEM images were used to determine the average grain size. Importantly, SEM examination revealed that the average grain size was considerably greater than the crystallite size estimated by X-ray diffraction (XRD). The difference between grain size seen in SEM images and crystallite size determined by XRD can be explained by the fact that SEM images capture collections of agglomerated crystallites, while XRD provides information on individual crystallite size. SEM images show grains formed by the aggregation of several crystallites, resulting in larger than expected grain sizes [35,38].

### 3.3 Optical studies

**Fig. 6:**
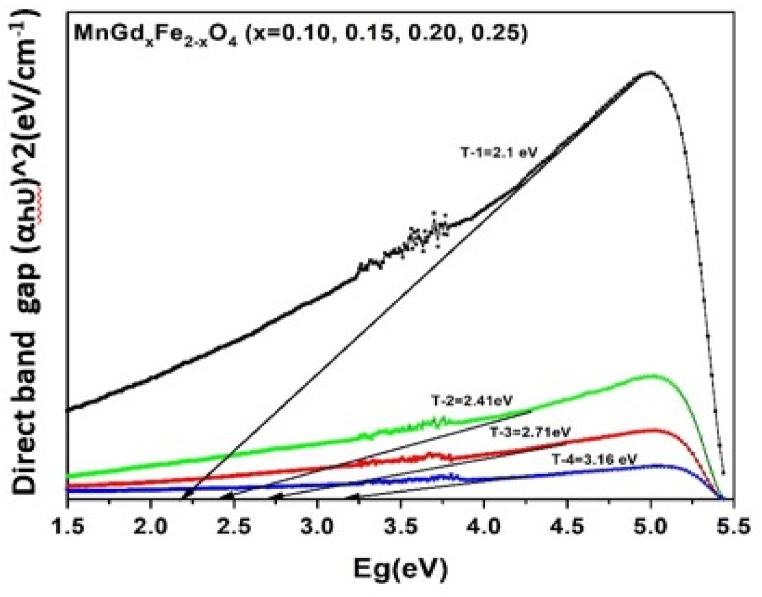
Illustrate the Band gap of Gd^3+^ doped MnGd_x_Fe_2-x_O_4_.

The wavelength of absorption reduces as the bandgap energy value rises and vice versa. The observed results, which are provided in Fig. 6, made it clear that with increasing dopant concentration of Gd^3+,^ the optical band gap widens, which increases the crystallite size. This suggests that the flaws in the spinel structure, which cause micro strain, have a similar relationship between crystal size and optical band gap energy [[39], [40], [41], [42]]. The Gd^3+^ and Fe^3+^ ions ionic radii as a result, increasing the optical bandgap energy of spinel nano ferrites. The ionic radii of Fe^3+^ ion with small radius (0.69 Å) by larger radius Gd^3+^ ion (1.078 Å) at B-site, which equilibrates the stress on B-site causing the observed change in crystal lattice. The SEM images was showed the porosity increased. According to published research, the optical bandgap energy values of pure Mn–Cu nano ferrites fall between 1.7 and 2.81 eV. However, adding rare-earth gadolinium ions to manganese nano ferrites causes a blue shift (2.1–3.16 eV) in the nano ferrites absorption band. This means that the synthesized nano ferrites might find use in microwave devices.

### 3.4 Magnetic property

**Fig. 7:**
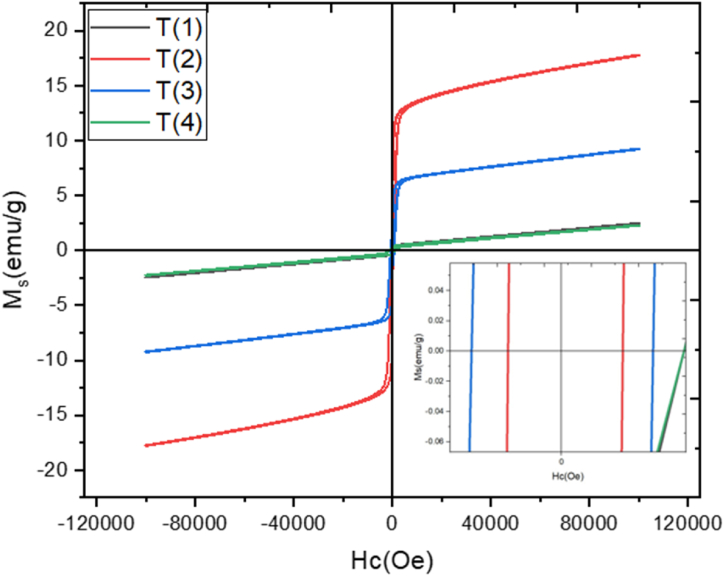
Graphs plotted applied field (T) versus magnetization (emu/g).

**Fig. 8:**
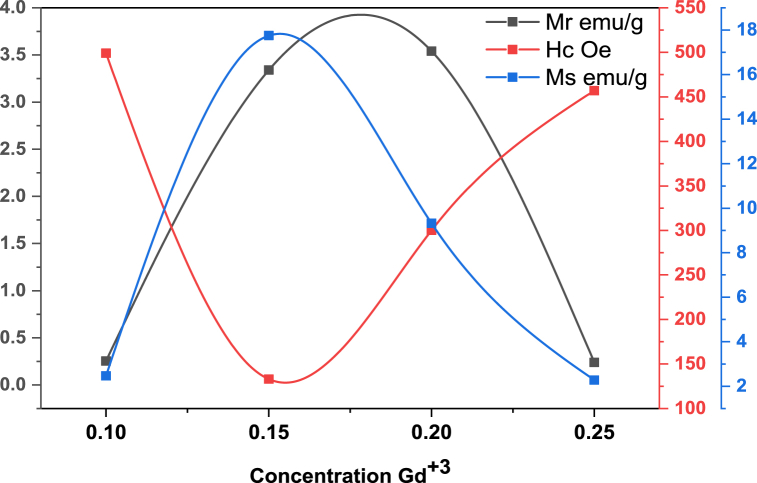
Plotted graphs concentration Gd ^3+^ versus H_c_, M_r_ and M_s_.

**Fig. 9:**
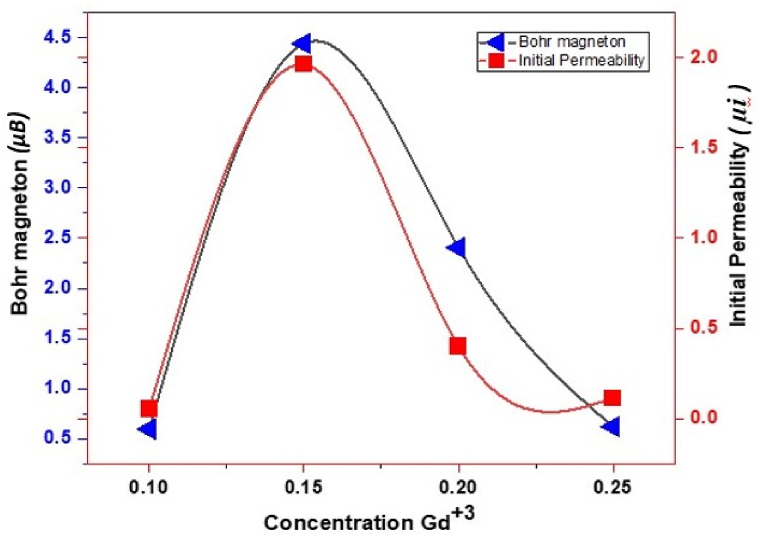
Plotted graphs concentration Gd^3+^ versus initial permeability and Bohr magneton.

Fig. 7 showed applied magnetic field versus magnetization(emu/g) at room temperature. In which explored magnetic properties of prepared spinel ferrites (MnGd_x_Fe_2-x_O_4_) at various concentration (x = 0.10, 0.15, 0.20, 0.25). As the magnetic field applied the coercivity, retentivity and saturation of magnetization gradually change and the magnetic hysteresis. Moreover, the magnetization saturation (M_s_), retentivity (M_r_), squareness ratio, coercivity (H_c_), initial permeability, and Bohar magneton were also calculated and shown in Fig. 8, Fig. 9. As the concentration of rare earth elements increased the retentively and saturation of magnetization also increased but abruptly these two factors decreased as the concentration increased. On the other hand, coercivity (H_c_) was increased at x = 0.10 as the concentration increased x = 0.15 the value of coercivity decreased as the content increase the coercivity also abruptly increased shown in Fig. 8. So, this nonlinear behavior shows a major effect of manganese with rare earth element Gd^3+^. Meanwhile, the hysteresis loop or graph was very simple and showed soft magnetic materials because coercivity values are very low as compared to hard ferrites [42,43]. In Fig. 9 the Bohar magneton and initial permeability were role-play similar to saturation of magnetization and retentivity. At x = 0.10 the values of Bohar magneton and initial permeability were very small as the contents increased these values increase and at x = 0.15 the rare earth Gd^3+^ values attain a high point so at x = 0.20 and 0.25these values were abruptly small. The magnetic properties of ferrites may be explained using Néel's two-sub-lattice ferrimagnetism model and the cation distribution between the A and B sites. The A-B super-exchange interactions, according to this model, are more important than the intra-sublattice A-A and B–B couplings. The difference in magnetic moments between sublattices B (octahedral) and A (tetrahedral) determines the resulting magnetic moment. The saturation of magnetization (M_s_) is (2.3–17.8) emu/g. The coercivity was calculated to range from 133 Oe - 499 Oe. The squareness ratio gives information to single-domain or multi-domain prepared material. But all values are <0.5 so synthesized nano ferrites may be the multi-domain structure. If square values have ≥0.5 so material may be a single domain structure. The above results/characteristics of MSF-NPs suggest for medical field [44,45].

Table 2 depict the saturation magnetization (emu/g), coercivity, retentivity and bohr magneton of sample T-1, T-2, T-3 and T-4. After analysis, it was explored that saturation magnetization occurs between 2.3 and 17.8 emu/g, coercivity range from 133 to 499 Oe, Retentivity range from 0.2 to 3.5 emu/g and Bohr magneton range from 0.596 to 4.441 μB as shown in above Table 2.

**Table 2:** VSM analysis depicts Saturation of magnetization, Coercivity, Retentivity, Squareness ratio, Initial permeability and Bohr magnetons.

Sample code	Saturation magnetization (*M*_*s*_) (emu/g)	Coercivity (*H*_*c*_) (Oe)	Retentivity (*M*_*r*_) (emu/g)	squareness ratio (*M*_*r*_*/M*_*s*_)	Initial permeability (*μi*)	Bohr magneton (*μB*)
**T-1**	2.5	499	0.3	0.102	0.053	0.596
**T-2**	17.8	133	3.3	0.188	1.961	4.441
**T-3**	9.3	300	3.5	0.379	0.400	2.406
**T-4**	2.3	457	0.2	0.104	0.114	0.620

### 3.5 Magnetic hyperthermia experiment

**Fig. 10:**
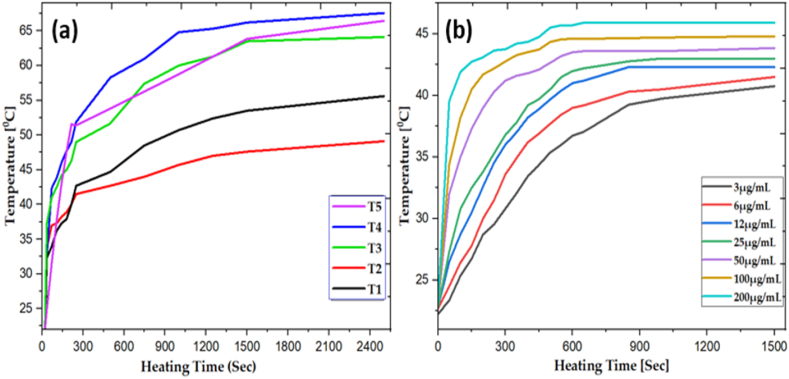
Shows the **(a)** Temperature Vs Time response of samples T1, T2, T3, T4 and T5, **(b)** Temperature Vs Heating Time of Sample T2 at various concentration.

In the magnetic Hyperthermia Experiment (MHE), The self-heating properties of the samples were evaluated under an alternating magnetic field of 30 mT in deionized water, while maintaining a frequency of 850 KHz for different samples (T1, T2, T3, T4, and T5). The temperature versus time responses for samples T1, T2, T3, T4, and T5 are shown in Fig. 10 (a). Initially the temperature increased rapidly until it reached the Curie temperature, after which it remained constant. These results provide strong evidence that the sample performs well at high frequencies due to its rapid thermal response. Therefore, all samples exhibit a good heating curve, but sample T2 shows a particularly promising heating result.

Furthermore, we have performed experiment on sample T2 at different concentrations with keeping constant frequency, which are shown in Fig. 10 (b). The T2 sample exhibits a significantly higher saturation magnetization and a lower curie temperature. As it was expected that with higher particle concentrations, the dispersion sample would reach saturation temperature faster and achive higher temperatures value as compared to rest of the samples. The T2 sample, with concentrations ranging from 3 to 100 μg/mL, only achieved temperatures of 40.75, 41.5, 42.3, 43, 43.85 and 44.75 °C respectively, which were lower than the desired temperature of 46 °C for magnetic hyperthermia [46]. Morever, the T2 sample at 200 μg/mL concentration reached at temperature close to 45.57 °C. Interestingly, the saturation temperature for samples with high particle concentrations up to 200 mg/mL did not significantly increase with increasing particle concentration in the dispersion. Above curie temperature, increasing the concentrations of sample, a little bit increase in temperature. This behavior can be attributed to the lower curie temperature of the particles. Such heating characteristics of the particles make them suitable for controlled hyperthermia [47]. The optimal particle concentration for hyperthermia at the cancer site falls between 100 and 200 mg/mL, resulting in a temperature increase of the site to 46 °C. Even if the particles accumulate locally at concentrations exceeding 120 mg/mL, the temperature of the site does not exceed 47 °C, which is the threshold for potential damage to normal tissues due to excessive heating. Therefore, by utilizing T2 sample (MSF-NPs), we can achieve controlled hyperthermia without the risk of overheating.

### 3.6 Specific absorption rate

The heating capacity of MSF-NPs was determined by measuring the specific absorption rate (SAR), which represents the rate at which heat is dissipated per unit mass of MNPs. This rate is influenced by three material properties, including hysteresis losses, Néel relaxation times (associated with particle relaxation), and Brownian relaxation times (related to spin). The reported studies have shown that ferromagnetic MNPs with exchange coupling between phases or hard magnetic phases exhibit higher SAR values [[48], [49], [50], [51]]. However, in the field of nanomedicine, superparamagnetic and soft magnetic phase MNPs are preferred over ferrimagnetic and hard magnetic phase MNPs due to their tendency to promote aggregation and thrombosis [52,53].

The specific absorption rate (SAR) values can be calculated by this Eq. (8).

(8) $$Specific\;Absorption\;Rate\;\left(SAR\right)=\frac{C_{P\;water}}{m_{MSF-NPs}}\times \frac{dT}{dt}$$

Here, C_p water_ represents the heating capacity of water, The concentration of MSF-NPs is denoted as m_MSF-NPs_, and dT/dt signifies the rate at which temperature changes over time.

### 3.7 Anticancer activity

**Fig. 11:**
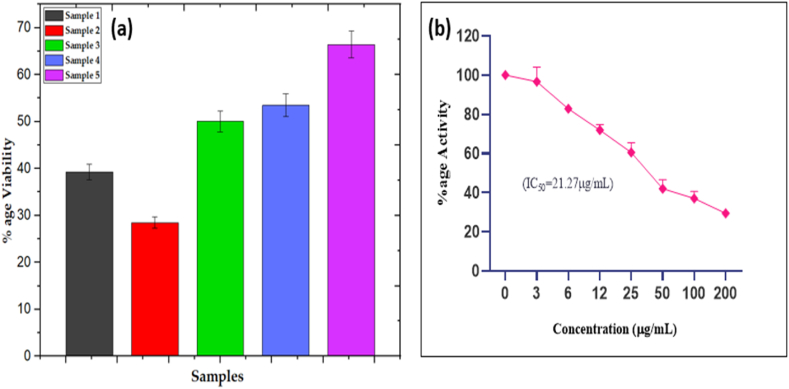
Shows **(a)** Loss of (%Cell Viability) samples T1, T2, T3, T4 and T5, **(b)** the %Cell Viability loss Vs Concentration (μg/ml) of sample T2.

The MTT assay was used to determine the inhibitory potential of MnGd_0_Fe_2_O_4_, MnGd_0.1_Fe_1.90_O_4_, MnGd_0.15_Fe_1.85_O_4_, MnGd_0.2_Fe_1.80_O_4_ and MnGd_0.25_Fe_1.75_O_4_ MSF-NPs samples of various concentration of Gadolinium doped manganese Iron oxide nanocomposites against MCF-7 cells. In preliminary screening, the cytotoxicity activities of MnGd_0_Fe_2_O_4_, MnGd_0.1_Fe_1.90_O_4_, MnGd_0.15_Fe_1.85_O_4_, MnGd_0.2_Fe_1.80_O_4_ and MnGd_0.25_Fe_1.75_O_4_ MSF-NPs samples were investigated at 200 μg/mL, and the obtained results are presented in Fig. 11(a). From this screening, significant inhibition was observed in samples MnGd_0_Fe_2_O_4_ and MnGd_0.1_Fe_1.90_O_4_ MSF-NPs compared to three other samples. But, the MnGd_0.1_Fe_1.90_O_4_ MSF-NPs showed >70 % inhibition against MCF-7 cells. The sample MnGd_0.1_Fe_1.90_O_4_ (T2) exhibits reasonably higher saturation magnetization and more favorable coercivity for MHT treatment. These results were further validated for antiproliferative activity of MnGd_0.1_Fe_1.90_O_4_ MSF-NPs sample in a dose dependent manner (3, 6, 12, 25, 50, 100 and 200 μg/mL) and dose-dependent curve was obtained, shown in Fig. 11(b). The IC_50_ value was calculated 21.27 μg/mL showing the strong inhibitory activity of these nanoparticles. This data showed that MnGd_0.1_Fe_1.90_O_4_ MSF-NPs can serve as a starting point for the further identification and isolation of MCF-7 cells inhibitory compounds or development of anticancer functional foods [54].

### 3.8 Magnetic resonance imaging (MRI)

**Fig. 12:**
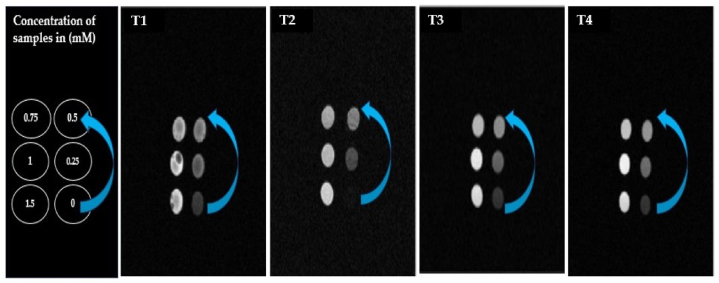
(T1, T2, T3 and T4). Shows T1 bright contrast MR images of MSF-NPS at (0, 0.25, 0.5, 0.75, 1 and 1.5 Mm).

Magnetic resonance imaging using a 1.5T scanner acquires Gadolinium (Gd) doped iron oxide (Fe_2_O_4_) nanoparticles and the result was calculated as the increase in Gd concentration, then the obtained results were significantly brighter, which showed that Gd provides a T1 bright contrast agent. In vitro and in vivo, these Magnetic Spinal ferrites nanoparticles (MSF-NPs) provide fantabulous T1 bright contrast agents for MRI. In the current study, low concentration of Gd doped Fe NPs exhibits less brightness than high of concentration Gd doped Fe_2_O_4_ NPs. Hence, high concentration Gadolinium (Gd) doped iron oxide nanoparticles can be used as a bright (T1) contrast agent for excellent results. Fig. 12(T1, T2, T3 and T4). shows T1-bright contrast MR images of MSF-NPs at different concentrations (0, 0.25, 0.5, 0.75, 1 and 1.5 Mm) [55,56]. In this discussion, Chen et al. [57] demonstrated that Gadolinium based iron oxide/manganese oxide NPs shows a excellent performance against cancer therapy and enhanced the T1 contrast agent. Marangoni et al. [58] reported that Gd-DOTA-SCN Chelate enhanced the MRI images and delivers a high relaxitivity r_1_ = 22 to 24 mM^−1^ S^−1^ as compared to Gd-DOTA r_1_ = 2 to 3 mM^−1^ S^−1^, provide efficient results against multi-biomedical imaging and diagnostic application. Lv et al. [59] investigated that facile synthesized PVP-coated GdIO NPs were used for small size MRI quantification, biodistribution MNPs and showed improved T1 bright contrast property. Recent studies also show that increasing the concentration of Gadolinium (Gd) enhances MRI as a T1 bright contrast agent because Gd itself a T1 contrast agent [60].

## 4 Conclusion

The magnetic spinal ferrites nanoparticles (MSF-NPs) were fabricated by sol-gel auto combustion method. The crystal structure confirmed via x-ray diffraction technique. The lattice constants were range (8.29–8.34 Å). The x-ray density ranged from 5.6 to 5.9 g/cm^3^. The bulk density has range from 2.3 to 2.8 g/cm^3^. The average crystalline size range from 11.3 nm to 23.9 nm. The morphology of the fabricated materials is porous, agglomerated spherical grains and non-uniform surface was observed. The activation energy has range from 0.495 to 0.423 eV. The magnetic properties were studied by VSM technique. The IC_50_ = 21.27 μg/ml value was calculated by using MTT assay showing the strong proliferation and inhibitory activity of these nanoparticles. These results suggested that the developed MSF-NPs are much suitable for remote-controlled hyperthermia therapy for cancer treatment, drug delivery, and MRI application due to their excellent magnetic properties and the 2nd reason is that the superparamagnetic and soft magnetic phase MNPs are preferred over ferrimagnetic and hard magnetic phase MNPs due to their tendency to promote aggregation and thrombosis. Hence, MSF-NPs could be beneficial source for cancer treatment. This study could play a dynamic role in medical science, nanomedicine, especially in cancer diagnosis like (MRI, CT, etc.) and combined therapy in the near future. To the best of our knowledge, the novelty of the research is that it is the first study to validate/demonstrate the anticancer and magnetic resonance imaging (MRI) efficacy of magnetic spinel ferrite nanoparticles (MSF-NPs). We recommend further in vivo anticancer such as (controlled Hyperthermia therapy, drug delivery) and MRI studies of magnetic spinal ferrite nanoparticles (MSF-NPs).

## Ethical approval and consent to participate

Cells/in vitro experimental study was conducted according to animal/cell guidelines accordance with ethical standard and monitored and approved by ethics review committee, Department of microbiology, faculty of life sciences, Government College University Faisalabad (GCUF), Punjab, Pakistan Ref. No. GCUF/ERC/24, Date: December 03, 2021.

## Consent for publication

Not applicable.

## Data availability statement

Data will be made upon reasonable request.

## Additional Information

No additional information is available for this paper.

## CRediT authorship contribution statement

**M. Tahir:** Software, Methodology, Conceptualization. **M. Fakhar-e-Alam:** Writing – review & editing, Supervision, Project administration. **Muhammad Asif:** Writing – original draft, Visualization, Supervision, Data curation, Conceptualization. **M. Javaid Iqbal:** Resources. **Aoun Abbas:** Methodology. **Mudassir Hassan:** Writing – review & editing. **Javed Rehman:** Writing – review & editing. **Qaisar Abbas Bhatti:** Writing – review & editing. **Ghulam Mustafa:** Writing – review & editing. **Asma A. Alothman:** Writing – review & editing, Funding acquisition. **Saikh Mohammad:** Writing – review & editing, Funding acquisition.

## Declaration of competing interest

The authors declare that they have no known competing financial interests or personal relationships that could have appeared/looked to influence the work reported in this paper.
