# Supplementary material for: Investigation of gadolinium doped manganese nano spinel ferrites via magnetic hypothermia therapy effect towards MCF-7 breast cancer
Source: Heliyon. 2024 Jan 19;10(3):e24792. doi: 10.1016/j.heliyon.2024.e24792 (PMC10837566; doi:10.1016/j.heliyon.2024.e24792)
Supplement: Multimedia component 1 [file mmc1.docx]

**Supplementary File/Data**


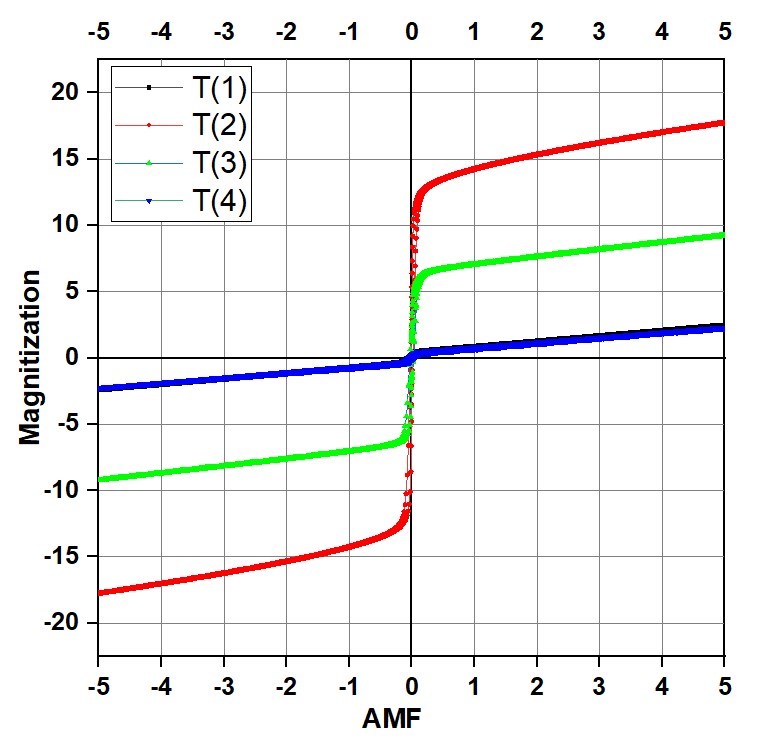


**Figure 1S:** VSM images of synthesized Samples

**Figure 2S:** %Cell viability graph of synthesized Samples


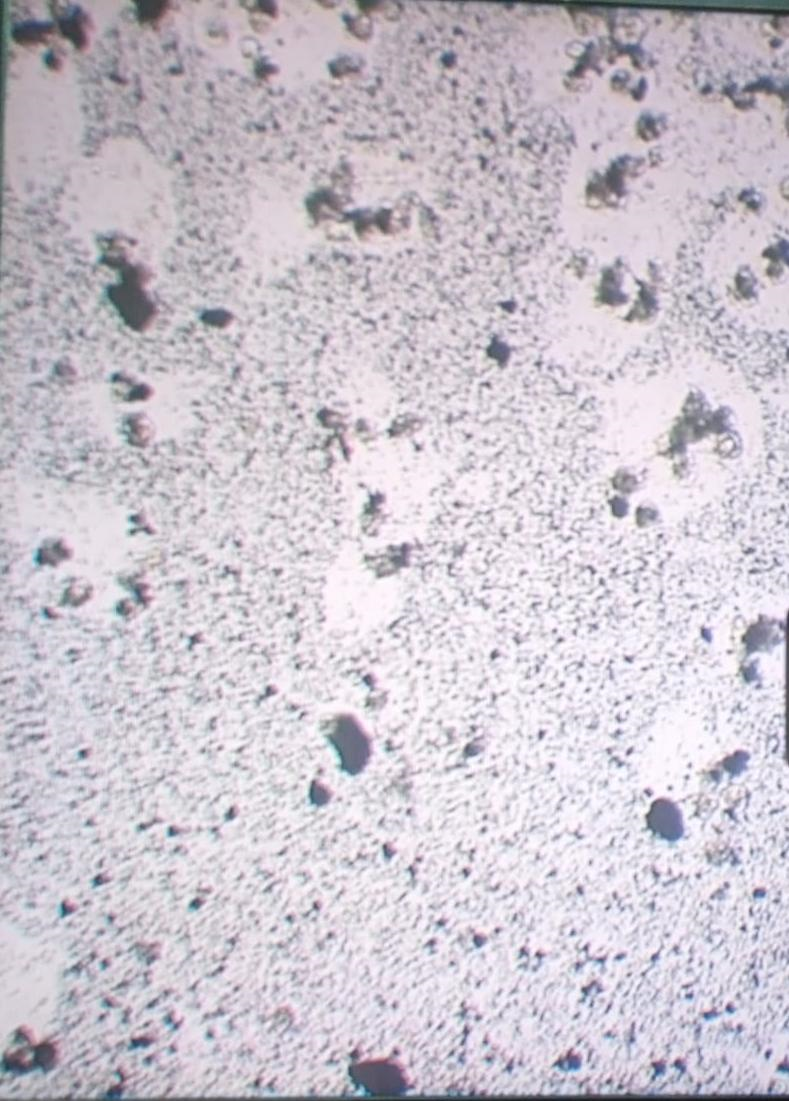


**Figure 3S:** MCF-7 cells culturing image of synthesized Sample, T1 and T3 at 1µm


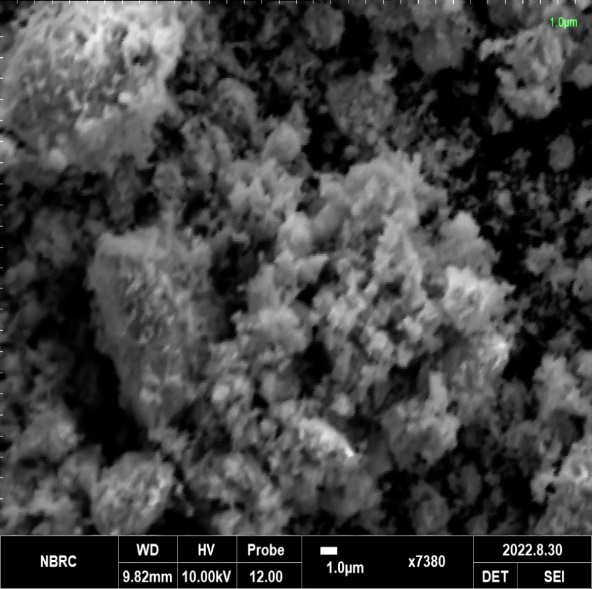

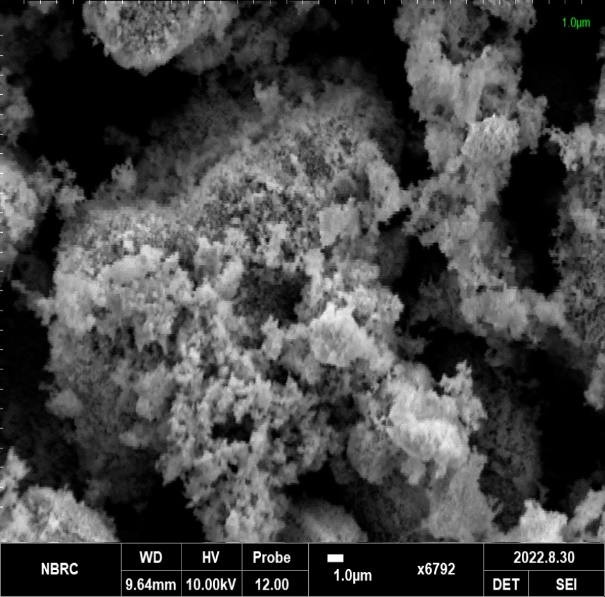


**Figure 4S:** SEM images of synthesized Sample, T1 and T3 at 1µm


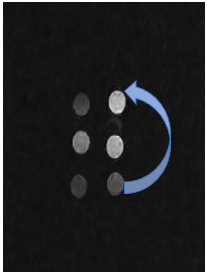


**Figure 5S:** MRI image of synthesized T1 Sample at /unknown concentration solution
